# Vaginal Bleeding in a Peri-Menopausal Woman

**DOI:** 10.24908/pocus.v9i1.16621

**Published:** 2024-04-22

**Authors:** Ambika Shivarajpur, Brian Kohen

**Affiliations:** 1 Department of Emergency Medicine, Memorial Hospital West Pembroke Pines, FL USA

**Keywords:** POCUS, molar pregnancy, snowstorm appearance, hydatidiform mole, complete molar pregnancy, partial molar pregnancy Presentation

## Abstract

Point of care ultrasound (POCUS) is a useful modality to initially identify a molar pregnancy. In this case, we describe a 51-year-old perimenopausal woman who presented to the emergency department (ED) with vaginal bleeding. A transvaginal POCUS was performed, revealing findings concerning for a molar pregnancy. These findings led to prompt diagnosis and treatment.

## Presentation

**Figure 1:**
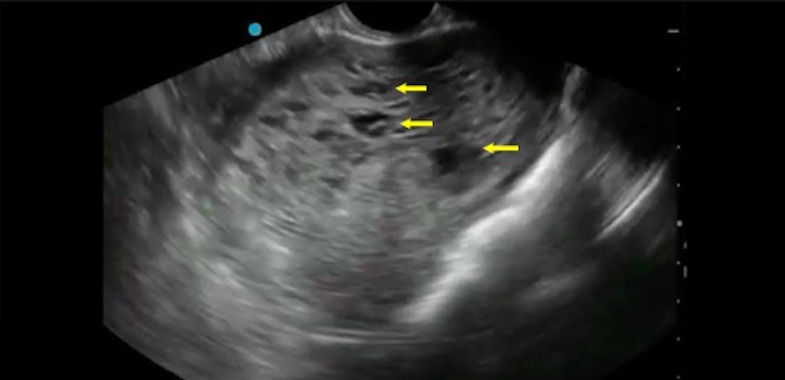
Transvaginal POCUS. Transverse view of an echogenic, enlarged uterus with cystic lesions (arrows) representing the classic“snowstorm” appearance consistent with a complete hydatidiform mole

A 51-year-old peri-menopausal woman with no significant past medical history presented to the ED with painless vaginal bleeding for 1 day. Her vital signs were within normal limits. On physical examination, the patient had a minimal amount of blood in the vaginal vault. She had no adnexal tenderness to palpation or active bleeding. A transvaginal POCUS revealed an enlarged uterus with cystic, hypoechoic lesions, prompting suspicions of a molar pregnancy (Figure 1, Video S1).

Evaluation of the adnexa on transvaginal ultrasound was unremarkable. This finding prompted the ED provider to obtain a beta-human chorionic gonadotropin level, which returned greater than 350,000 mIU/mL (< 5 mIU/mL). The patient remained hemodynamically stable and underwent suction curettage by gynecology as well as pathological examination of the uterine contents. The findings showed hydropic villi, and proliferation of cytotrophoblasts and syncytiotrophoblasts. These confirmed a complete molar pregnancy.

## Discussion

A hydatidiform mole, also known as a “molar pregnancy”, is an abnormal pregnancy characterized by placental villi with focal swelling, trophoblastic proliferation, and reduplication of genetic material. Hydatidiform moles are distinguished as either complete or partial moles 1. A complete mole is paternally derived, where sperm fertilizes an enucleated ovum 2. Duplication of a haploid sperm results in genotype 46, either XX or XY 2. In contrast, when sperm fertilizes a normal, nucleated ovum, this results in a partial mole with genotype 69, XXY 1, 2. Molar pregnancies occur in approximately 0.5-2 pregnancies per 1,000 2. The most common presenting symptom is vaginal bleeding 3. Risk factors for a molar pregnancy include extremes of ages (<21 or >35 years-old), previous history of molar pregnancy, and nulliparity 2. Generally, molar pregnancies are not viable, and treatment includes molar evacuation 2. The definitive diagnosis of molar pregnancies is made by histological evaluation, which is not immediately available. Given that approximately 3% of hydatidiform moles progress to choriocarcinoma 2, prompt diagnosis and treatment of a molar pregnancy is imperative.

Ultrasound has been used as an initial screening modality for detection of molar pregnancy given its accessibility, accuracy, and rapid identification of key features consistent with the diagnosis 4. Transvaginal ultrasound is more commonly used due to the higher frequency of the endocavitary probe compared to the curvilinear probe used for transabdominal examinations, allowing for a higher resolution image. In both POCUS and comprehensive transvaginal ultrasound, the uterus is viewed in both a transverse and sagittal orientation. It is often requested that the patient empty their bladder prior to scanning as when the bladder is full, it may obscure evaluation of the uterus. Abnormal sonographic findings in molar pregnancy include a focal cystic space within the placenta in patients with partial moles, or an enlarged uterus with multiple hypoechoic cystic lesions in patients with complete molar pregnancy. The classic description of complete molar pregnancy on ultrasound is a “snowstorm” appearance of the uterus due to the numerous hypoechoic cystic lesions present. In this case of complete molar pregnancy, the use of transvaginal POCUS led to prompt diagnosis and treatment.

## Disclosures

All authors report no disclosures related to this work.

## Patient Consent

The authors obtained informed consent from the patient. The patient gave consent to use de-identified images, videos, and health information for the purpose of publishing in this scientific journal. 

## Supplementary Material

 Video S1 Transvaginal POCUS demonstrating an enlarged uterus with cystic lesions consistent with a complete molar pregnancy.
